# Supplementary material for: Sensor-as-a-Service: Convergence of Sensor Analytic Point Solutions (SNAPS) and Pay-A-Penny-Per-Use (PAPPU) Paradigm as a Catalyst for Democratization of Healthcare in Underserved Communities
Source: Diagnostics (Basel). 2020 Jan 1;10(1):22. doi: 10.3390/diagnostics10010022 (PMC7169468; doi:10.3390/diagnostics10010022)
Supplement: Supplementary file 1 [file diagnostics-10-00022-s001.pdf]

diagnostics

Supplemental Section (S1)

1. Biosensor Literature Search

The objective of this search was to determine the number of *E. coli* biosensors that have been published in peer-reviewed journals in the past ten years and to screen for key terminology related to the degree of suitability of the biosensors for applications in real-world settings.

2. Approach

The search engine *Web of Science* (Clarivate Analytics, United States) was used for literature search and analysis. Using the *advanced search* tool, two key words were combined using the following Booleans and field tags:

TI = (E. coli "OR" Escherichia coli)

TS = (Biosensor)

The results of this search were restricted to show only research articles published between 2009 and 2019.

To screen for the papers that include assertions related to suitability for applications in real-world settings, an additional TS field tag was included with one of the following claims: tested in real samples, low-cost, user-friendly, portable, real-time analysis.

3. Results

The first round of screening yielded a total of **453** research articles containing the word *E. coli* or *Escherichia coli* in the title and the word biosensor within the manuscript. A manual screening was further conducted to remove all publications that were not directly related to the topic of biosensors for *E.coli* detection, resulting in a total of **303 articles.** Tables S1 and S2 show the number of research articles published every year from 2009 to 2019, and the top funding agencies involved in *E. coli* biosensors research respectively.

Table S1. Number of research articles published every year for the past ten years in peer-reviewed journals on the topic of *E. coli* biosensors.

Year	Number of Articles
2019	32
2018	42
2017	43
2016	33
2015	39
2014	19
2013	29
2012	21
2011	26
2010	10
2009	9

Table S2. Top five agencies that provide funding for research on *E. coli* biosensors.

Funding Agencies	Number of Articles
National Natural Science Foundation of China	69
Natural Sciences and Engineering Research Council of Canada	13
United States Department of Agriculture (USDA)	10
National Science Foundation (NSF)	9

Science Technology Commission of Shanghai Municipality (STCSM)	7

Another round of screening was conducted to elucidate the degree of suitability of the published biosensors for applications in real-world settings. Table S3 depicts the incidence of claims related to this issue.

Table S3. Depiction of research articles on *E. coli* biosensors that contain claims related to real-world applicability.

Claim	Number of Articles
Tested in Real Samples	88
Low-cost	31
User-friendly	6
Portable	26
Real-time Analysis	16
